# The Genetic Structure of the Romanian Male Population Revealed by 27 Y-Chromosome STR Loci

**DOI:** 10.3390/ijms27167121

**Published:** 2026-08-08

**Authors:** George Popoiu, Florin Stanciu, Paulina Popescu, Veronica Cuțăr, Simona Vladu, Violeta Nicola, Anastasia Procopciuc, Ana Rădulescu, Adnana Cotolea, Sergiu Emil Georgescu

**Affiliations:** 1 Doctoral School of Biology, Faculty of Biology, University of Bucharest, 050095 Bucharest, Romania; sergiu.georgescu@bio.unibuc.ro; 2 Department of Forensic Genetics, National Forensic Institute, General Inspectorate of Romanian Police, 020123 Bucharest, Romania; podgoreanu.paula@yahoo.com (P.P.); veracutar@gmail.com (V.C.); simonavladu30@yahoo.ro (S.V.); nicolaionelavioleta@gmail.com (V.N.); procopciucanastasia@gmail.com (A.P.); ana.rradulescu@gmail.com (A.R.); adnanacojocariu33@gmail.com (A.C.)

**Keywords:** Y-STR, Romania, haplotype diversity, forensic genetics, Discrete Laplace, haplogroup, match probability (PM), population genetics

## Abstract

Y-chromosomal Short Tandem Repeats (Y-STRs) provide insight into forensic evidence interpretation and male population history. Romania’s position at the crossroads of Europe and Western Asia makes it a key reference population for both domains, yet existing Romanian Y-STR databases are limited in geographic coverage and marker number. We genotyped 928 unrelated Romanian males across all 41 counties and Bucharest at 27 Y-STR loci using the Investigator Argus Y-28 Kit, and modeled haplotype frequencies using the Discrete Laplace (DL) distribution (disclapmix v1.7.5, 17-locus panel; disclapmix2 v0.6.1, 27-locus panel). Discrete Laplace modeling revealed 12 and 14 optimal mixture components for the 17- and 27-locus models, respectively, with median match probabilities of 1 in 1.25 × 10^7^ and 1 in 5.8 × 10^12^, reflecting nearly six order-of-magnitude gain in evidential weight. Haplogroup analysis identified 16 Y-lineages; R1a, I2a, E1b1b, J2a, and R1b collectively represent ~80% of the population, and reflect Romania’s layered Steppe, Balkan, and Near Eastern ancestry, with subtle regional gradients between Moldavia, Transylvania, and Muntenia. This dataset provides validated forensic reference data and new insights into the patrilineal genetic structure of the Romanian population.

## 1. Introduction

Forensic genetics is a branch of forensic science that applies molecular biology principles and methods to generate and interpret genetic evidence from biological samples recovered in the course of criminal investigations, providing information such as identity, biological relationships, or the possible origin of a trace. The results must be reported and interpreted within the bounds of what the underlying data can support.

The most widely used forensic genetics analyses are those that highlight a series of autosomal Short Tandem Repeat (STR) markers because they generate a genetic profile that is theoretically unique for each individual (except for monozygotic twins).

Even though this type of analysis offers numerous advantages and continues to be the gold standard in forensic genetics, there are also situations in which its efficacy is often challenged in forensic scenarios involving complex mixtures—most notably in sexual assault investigations where a high concentration of female deoxyribonucleic acid (DNA) can mask a trace amount of male genetic material. In these situations, the use of Y-chromosomal Short Tandem Repeats (Y-STRs) allows for the selective amplification of male DNA [1].

The forensic utility of Y-STRs arises not only from their ability to provide genetic information in male-dominant cases but the unique inheritance pattern of the Y-chromosome—passed almost entirely unchanged from father to son—grants it a distinct role in genealogical research, familial searching, and paternal lineage analysis [2]. This patrilineal transmission allows forensic geneticists to link unidentified remains to paternal relatives across multiple generations or to trace geographical ancestry through Y-haplogroups. However, the lack of recombination presents a unique statistical challenge: because changes in the structure of these markers only occur in the case of mutational events, many of the males in a paternal lineage can share the same Y-STR haplotype.

On the other hand, Romania occupies a strategically important position at the crossroads of Europe and the Near East and reflects a complex history of population movements including Neolithic farmer migrations from Anatolia, Bronze Age expansions from the Eurasian steppe, and subsequent Slavic and other historic-era gene flow [3,4]. Also, the previous Y-STR studies on Romanian samples have been limited in geographic coverage or marker number [5,6,7,8,9,10].

In order to better assess the weight of evidence obtained as a result of Y-STR typing, we conducted a study of the Romanian population to determine the range of the frequencies of Y-STR haplotypes using the Discrete Laplace distribution model, the Y-chromosome haplogroup frequencies and the regional particularities among the three historical regions, Moldavia, Transylvania and Muntenia.

Prior Y-STR investigations in Romania were conducted on smaller, geographically restricted samples and with marker panels that predate the extended commercial kits currently in routine forensic use. The present study is the first to combine complete national geographic coverage—sampling from all 41 Romanian counties and the municipality of Bucharest—with the full 27-locus profile generated by the Investigator Argus Y-28 Kit.

## 2. Results

### 2.1. Data Characteristics

All 928 haplotypes were unique when all 27 Y-STR markers were used. A summary of the analyzed data can be found in Table 1.

**Figure 1:**
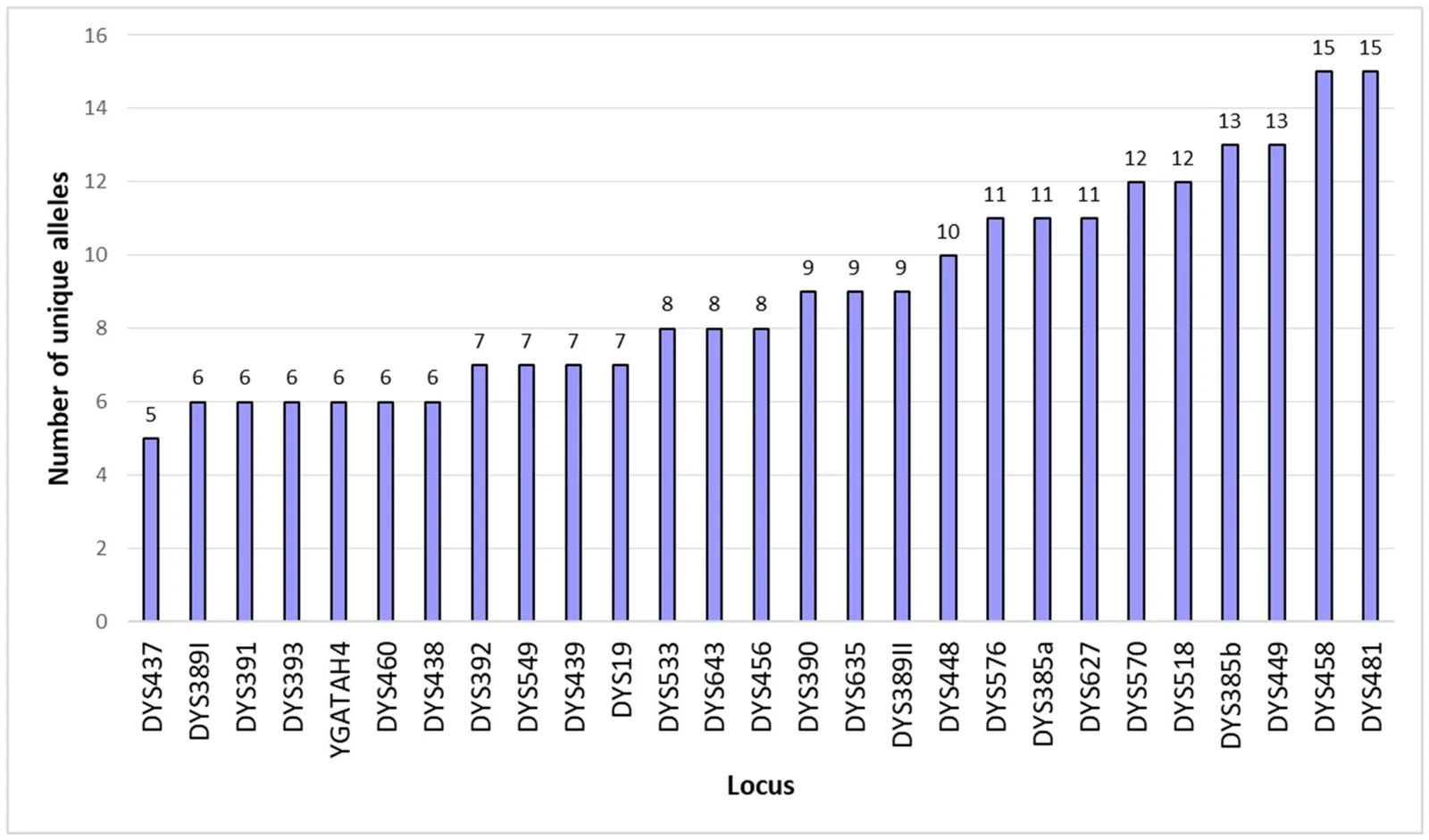
Distribution of Y-STR markers based on the number of unique alleles found.

The number of alleles for each marker analyzed (Figure 1) varies from 5 (for DYS437) to 15 (for DYS458 and DYS481) with a mean value of 9 alleles per locus and a total of 243 alleles found in the 928 samples analyzed.

**Figure 2:**
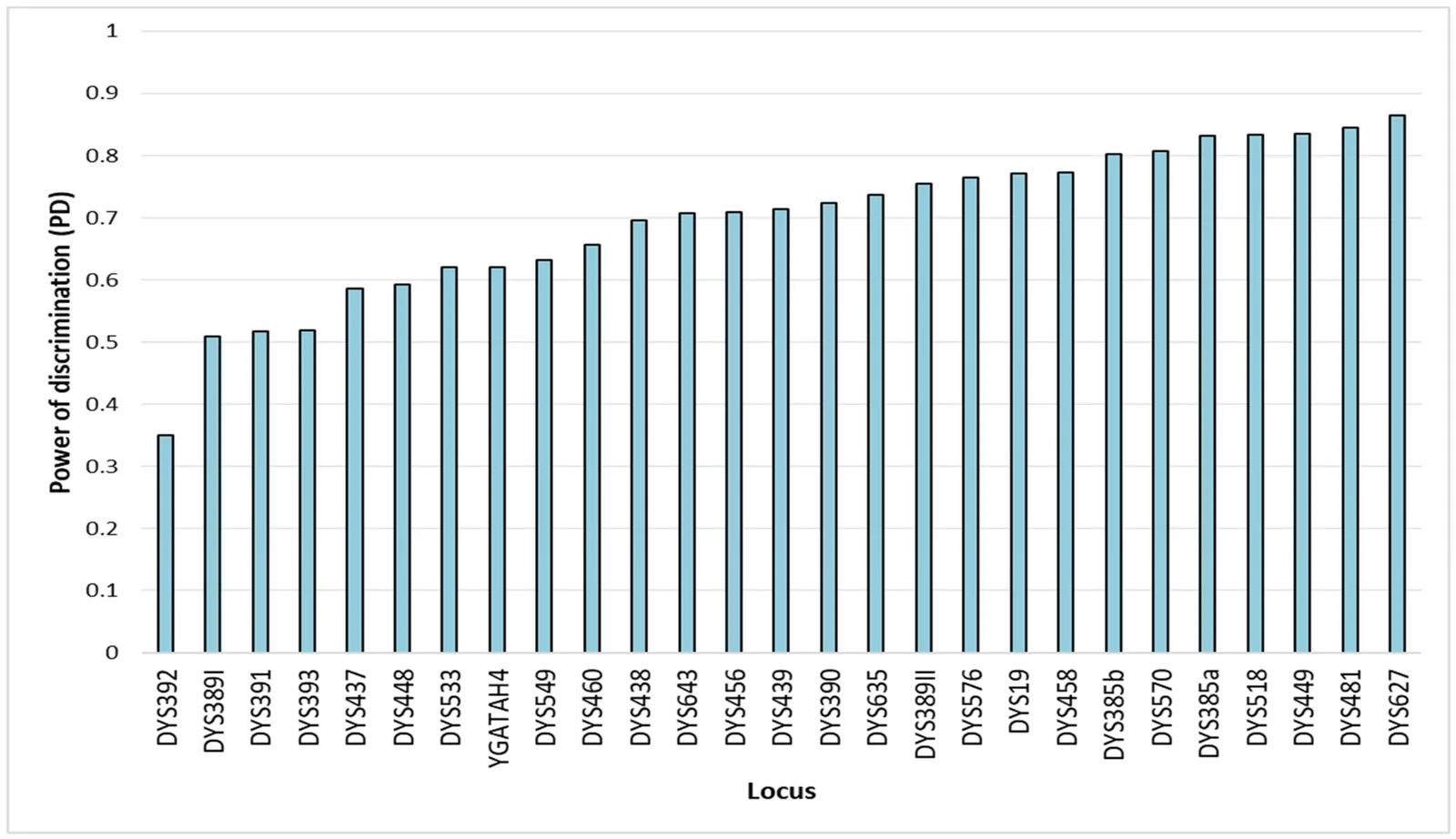
Distribution of Y-STR markers based on PD values.

As expected for haploid markers the values for power of discrimination (Figure 2) and gene diversity are similar, with the lowest values found at DYS392 marker (0.350 for PD and 0.351 for GD) and the highest at DYS627 (0.864 for PD and 0.865 for GD).

The data obtained also revealed that of the 27 Y-STR markers analyzed, 23 presented just full alleles and only four of them showed microvariants. In total, nine alleles with microvariants were observed, one each for the loci DYS390 (24.3), DYS437 (13.1), DYS448 (16.4) and six at the DYS458 locus (17.2, 18.2, 19.2, 20.2, 21.2, and 22.2). All nine designations are listed among previously cataloged alleles for these loci in the Y-Chromosome Haplotype Reference Database (YHRD, https://yhrd.org), and none represent a novel or previously unreported microvariant.

### 2.2. Discrete Laplace Analysis

#### 2.2.1. Using Disclapmix v1.7.5 [11,12]

Although all 928 haplotypes are unique when the full 27-locus profile is considered, the R package disclapmix v1.7.5 only supports haplotype frequency modeling for a defined 17-locus subset (DYS19, DYS389I, DYS389II, DYS390, DYS391, DYS392, DYS393, DYS437, DYS438, DYS439, DYS448, DYS456, DYS458, DYS635, YGATAH4, DYS385a and DYS385b). When the 928 haplotypes are considered at these 17 loci only, 899 distinct profiles are observed (872 singletons, 25 haplotypes shared by two individuals, and 2 haplotypes shared by three individuals), reflecting the reduced discriminatory power of the smaller marker panel rather than any exclusion of samples.

**Figure 3:**
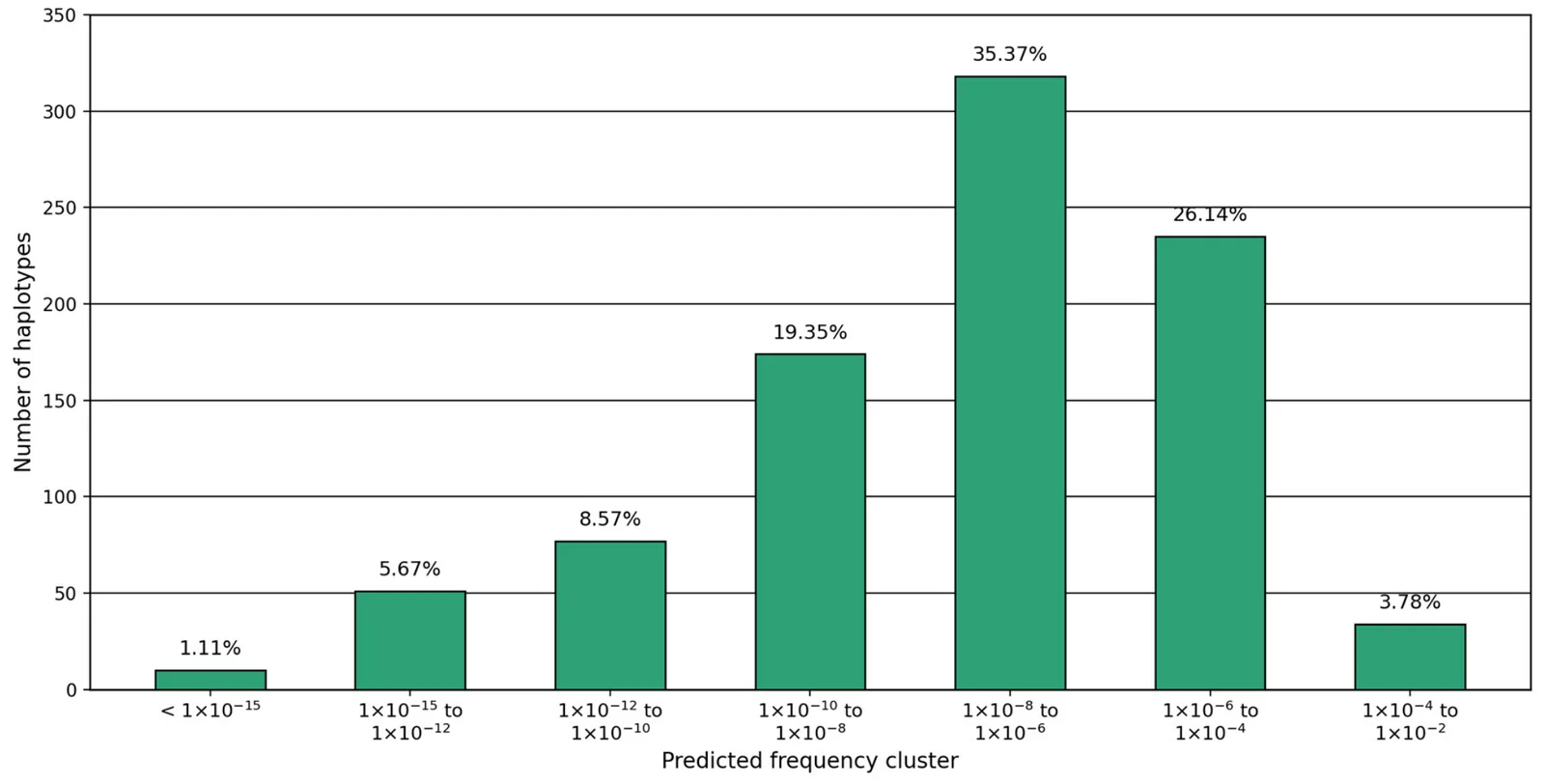
Haplotype count and percent per frequency cluster using disclapmix v1.7.5 (percentages are rounded to two decimal places and do not sum to exactly 100% due to rounding).

Mixture model analysis of the haplotypes using the Discrete Laplace distribution performed with disclapmix v1.7.5 yielded Bayesian Information Criterion (BIC) values that decreased from 47,293 at k = 1 to a global minimum of 34,192 at k = 12 clusters, before rising slightly and fluctuating for k > 12. The 12-cluster solution was therefore adopted as optimal. The BIC profile showed the steepest descent between k = 1 and k = 6, indicating that the first six clusters capture the bulk of haplotypic structure in the Romanian population. The predicted haplotype frequencies for the 928 haplotypes analyzed varied between 2.58 × 10^−18^ and 4.97 × 10^−3^, with the median value at around 8.01 × 10^−8^ (Figure 3).

The match probabilities computed for the 928 typed haplotypes varied between 1 in 2.01 × 10^2^ and 1 in 3.88 × 10^17^ with the median estimated match probability of approximately 1 in 1.25 × 10^7^.

#### 2.2.2. Using Disclapmix2 v0.6.1 [13]

Mixture model analysis using disclapmix2 v0.6.1 resulted in BIC values that decreased from 81,469 at k = 1 to a global minimum of 62,320 at k = 14 clusters, before rising slightly for k > 14. In the end, the 14-cluster solution was adopted as optimal.

**Figure 4:**
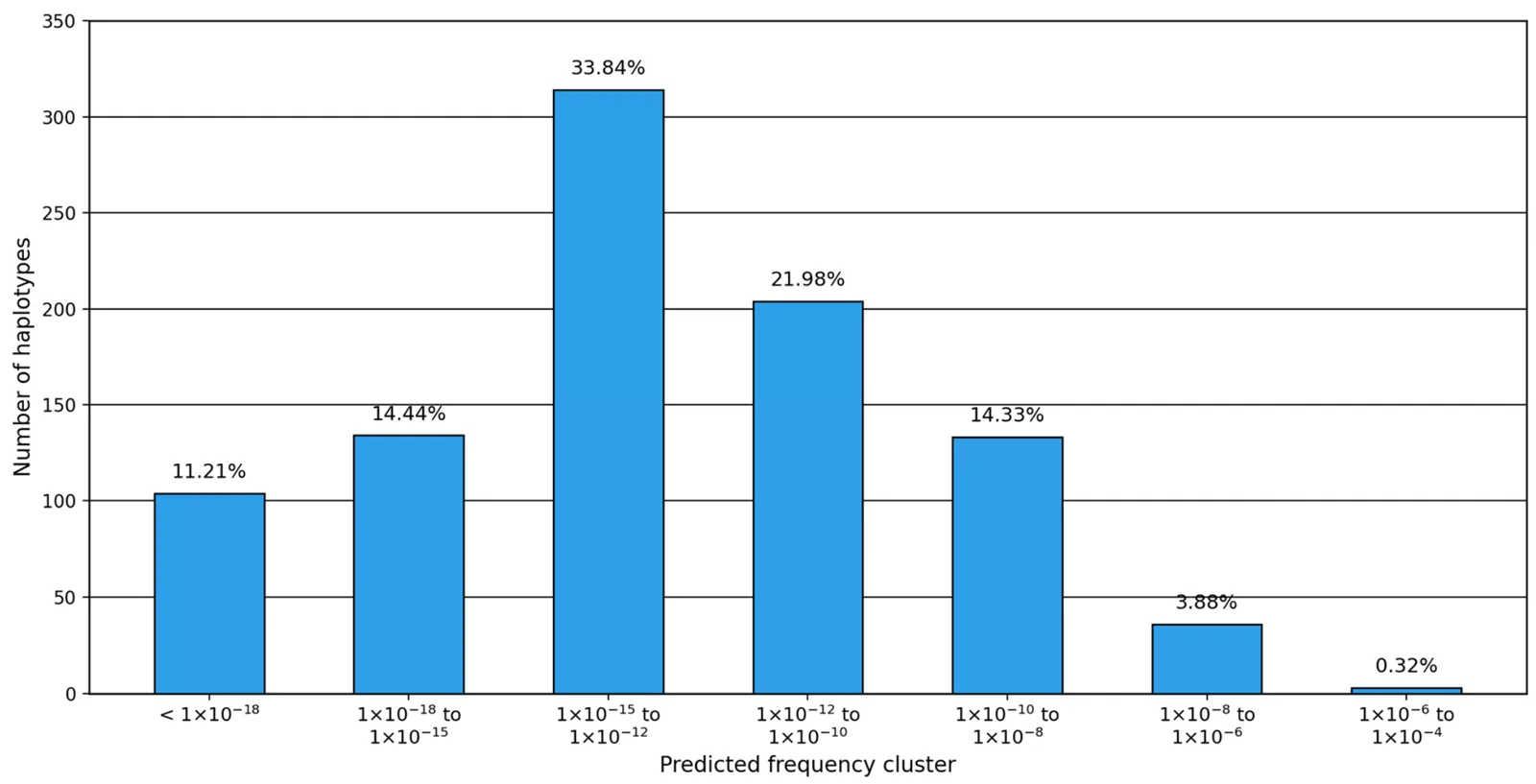
Haplotype count and percent per frequency cluster using disclapmix2 v0.6.1.

The predicted haplotype frequencies for the 928 haplotypes analyzed varied between 4.31 × 10^−28^ and 2.86 × 10^−6^, with the median value at around 1.73 × 10^−13^ (Figure 4).

For the disclapmix2 v0.6.1, the match probabilities values ranged from 1 in 3.50 × 10^5^ to 1 in 2.32 × 10^27^ with the median estimated match probability of approximately 1 in 5.8 × 10^12^.

The observed haplotype diversity was the same with disclapmix v1.7.5 and disclapmix2 v0.6.1 of 0.9989, but the model-based haplotype diversity differed between the two software packages, disclapmix v1.7.5 generating a value of 0.9272 while disclapmix2 v0.6.1 generated a value of 0.8292.

### 2.3. Y-Chromosome Haplogroup Diversity (HGD) Within the Romanian Population

Haplogroup assignment was successful for all 928 samples; 16 distinct haplogroups were identified. Supplementary Table S4 lists, for each of the 928 samples, the predicted haplogroup, the fitness score, and the Bayesian probability returned by Athey’s Haplogroup Predictor.

Table 2 presents, for the 16 distinct haplogroups identified, their absolute and relative frequencies for the total Romanian sample and the three regional subgroups.

At the national level, the five most frequent haplogroups were R1a (n = 188; 20.26%), I2a (n = 150; 16.16%), E1b1b (n = 142; 15.30%), J2a (n = 141; 15.19%), and R1b (n = 127; 13.69%), together accounting for 80.6% of the sample. This pentapartite haplogroup structure reflects the layered population history of Romania: R1a is the principal Steppe-ancestry lineage associated with Indo-European expansion [3,14]; I2a is the most dominant lineage in Balkan region which borders Romania to the south and with which there have been close historical ties [15]; E1b1b possibly represents migrations from North Africa and the Near East to Europe [16]; J2a reflects Anatolian and subsequent Near Eastern gene flow that are often associated with later Neolithic and Bronze Age movements into Europe [16,17]; and R1b which is associated with populations that migrated from the Eurasian Steppe into Europe like the Yamnaya culture [3,18]. Rarer haplogroups—including G2a (2.91%) [19], I1 (5.93%) [20], J2b (3.56%) [16], and N (1.51%) [21]—reflect additional Caucasian Neolithic, Northern European, Balkan-South Asian, and Uralic contributions, respectively.

**Figure 5:**
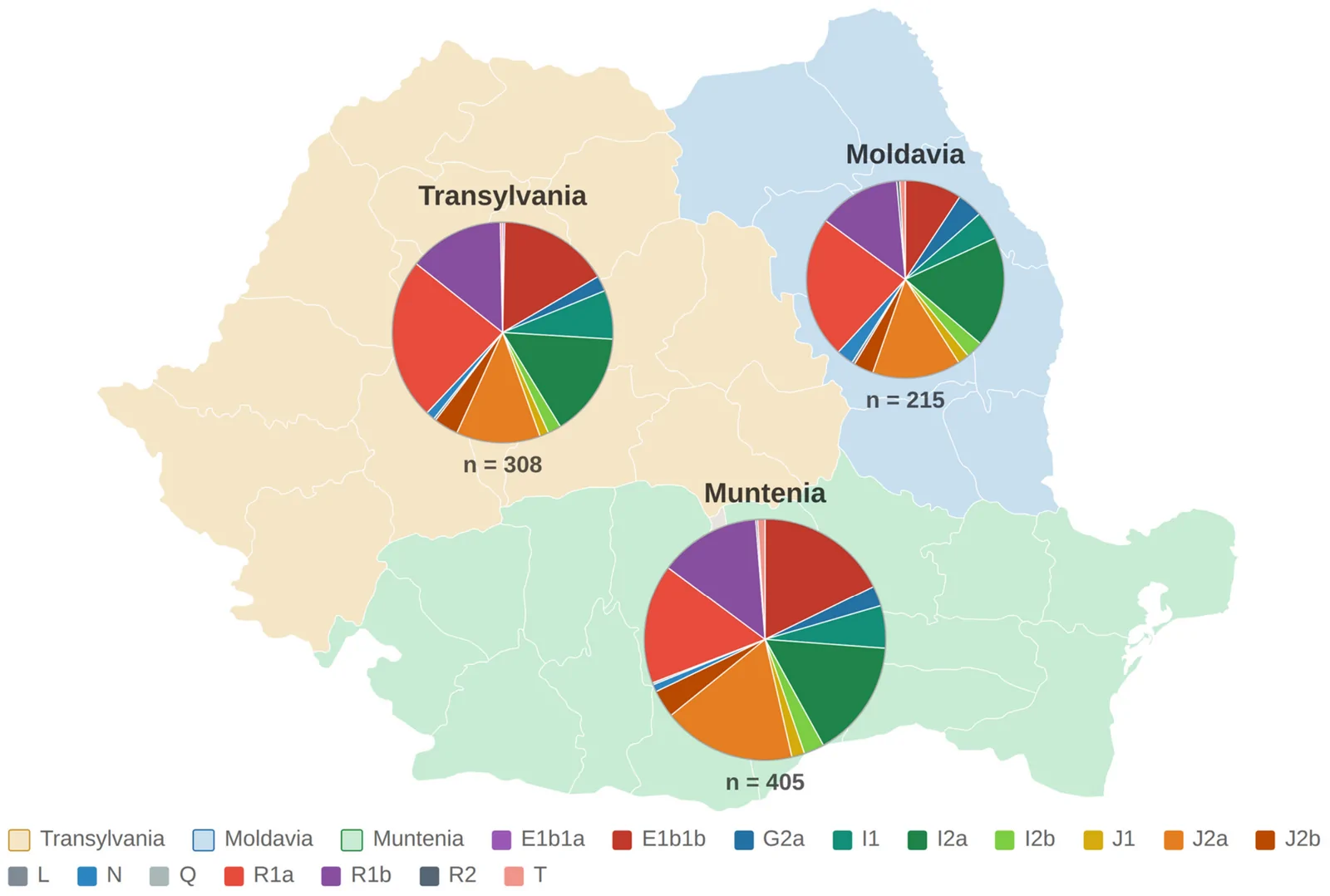
Y-chromosome haplogroup distribution map by region (Map created using DiMarco, M. Datamaps: Customizable SVG map visualizations for the web).

Regional comparisons (Figure 5) revealed that R1a was jointly the most frequent haplogroup in Moldavia (23.26%) and Transylvania (23.70%), while J2a shared the top position in Muntenia (17.78%, tied with E1b1b). Moldavia showed the lowest E1b1b frequency (9.30%) compared with Transylvania (16.23%) and Muntenia (17.78%). I2a was most prevalent in Moldavia (18.14%). N was more frequent in Moldavia (2.79%) than in Muntenia (0.99%).

To formally test whether these regional differences were statistically significant, an Analysis of Molecular Variance (AMOVA) was performed on the full 27-locus haplotypes using the R_ST_ estimator [22]. The global three-region AMOVA yielded R_ST_ = 0.0026, indicating that only 0.26% of the total molecular variance was attributable to differences among regions; this result was nonetheless statistically significant (10,000 permutations, *p* = 0.027). Pairwise comparisons showed that Moldavia and Muntenia differed significantly (R_ST_ = 0.0052, *p* = 0.009), whereas Moldavia versus Transylvania (R_ST_ = −0.0005, *p* = 0.527) and Transylvania versus Muntenia (R_ST_ = 0.0023, *p* = 0.062) did not reach significance. These haplotype-level results indicate that, although the Romanian male population is overall genetically homogeneous, a small but detectable degree of regional substructure exists, driven mainly by differentiation between the Moldavia and Muntenia subgroups.

Haplogroup diversity was high and similar across all three regions (Moldavia: HGD = 0.8584 ± 0.0338; Transylvania: HGD = 0.8518 ± 0.0291; Muntenia: HGD = 0.8610 ± 0.0248), with the national estimate at HGD = 0.8608 ± 0.0164, indicating that the haplogroup-level inter-regional differentiation is modest relative to within-region diversity.

Supplementary Table S1 is an Excel spreadsheet that contains the haplotypes used and the predicted frequencies with disclapmix v1.7.5 R package, and the match probabilities calculated based on the frequencies obtained.

Supplementary Table S2 is an Excel spreadsheet that contains the haplotypes used and the predicted frequencies with disclapmix2 v0.6.1 R package, and the match probabilities calculated based on the frequencies obtained.

Supplementary Table S3 is an Excel spreadsheet that contains the alleles observed and the count of them in the analyzed samples for all 27 Y-STR loci.

Supplementary Table S4 is an Excel spreadsheet that contains Athey’s haplogroup predictions, fitness score and probability of the haplogroup for all 928 samples analyzed.

Supplementary Figure S1 is an HTML file which features an interactive map showing the distribution of Y-STR based haplogroups by historical region.

Supplementary Figure S2 is a .tif file which features the haplotype percent per frequency cluster using disclapmix v1.7.5 in a pie chart format (percentages are rounded to two decimal places and do not sum to exactly 100% due to rounding).

Supplementary Figure S3 is a .tif file which features the haplotype percent per frequency cluster using disclapmix2 v0.6.1 in a pie chart format.

## 3. Discussion

The results demonstrate that using a larger number of Y-STR markers increases the chances of discrimination between individuals: using the 17 markers considered by disclapmix v1.7.5 out of the 928 haplotypes only 899 were unique, but when all 27 markers were considered as disclapmix2 v0.6.1 does, all 928 were unique.

Both methods correctly identify structured population substructure, but disclapmix v1.7.5 selects 12 clusters, while disclapmix2 v0.6.1 selects 14 clusters. The higher absolute BIC for disclapmix2 v0.6.1 is expected given that BIC scales with the number of parameters, and 27 loci with 14 clusters means 584 parameters versus ~243 for the disclapmix v1.7.5 model. The BIC improvement from k = 1 to the optimum is actually larger for disclapmix2 v0.6.1 (−19,149 vs. −13,101), indicating that the additional loci reveal more genuine genetic substructure.

The most consequential difference between the two methods used was observed in the predicted frequency values. The median predicted frequency shifts from 8 × 10^−8^ (disclapmix v1.7.5) to 1.7 × 10^−13^ (disclapmix2 v0.6.1)—nearly six orders of magnitude. In the disclapmix v1.7.5 analysis, 77.2% of haplotypes fall in the 10^−9^ to 10^−3^ frequency range, and no haplotypes fall below 10^−18^. In the disclapmix2 v0.6.1 analysis, 89.7% of haplotypes fall below 10^−9^, and 11.2% fall below 10^−18^—a range entirely inaccessible to the 17-locus model. This means that the forensic evidential weight of a haplotype match, expressed as the match probability, goes from “1 in 1.25 × 10^7^” to “1 in 5.8 × 10^12^” unrelated males. For matched haplotypes, disclapmix v1.7.5 frequencies are consistently about 10^4^–10^6^ × higher than disclapmix2 v0.6.1 values, which is the direct mathematical consequence of conditioning on 10 fewer loci.

The wide power-of-discrimination range observed across the 27 markers—from 0.350 at DYS392 to 0.864 at DYS627—confirms that the panel’s evidential contribution is not dominated by a handful of highly polymorphic loci but distributed across the marker set. This redundancy provides resilience against partial profiles, which are common in degraded or low-template casework samples.

As we stated before, the highest gene diversity and power of discrimination were observed at DYS627 (GD = 0.865; PD = 0.864). The elevated diversity at DYS627 is consistent with its classification as a rapidly mutating Y-STR [23]. This pattern extends across the panel: the six RM Y-STRs included in this kit (DYS449, DYS481, DYS518, DYS570, DYS576, DYS627) rank among the ten most diverse of the 27 loci studied (GD = 0.826 ± 0.035 vs. 0.659 ± 0.117 for the remaining loci), consistent with their markedly higher mutation rate relative to conventional Y-STRs.

To further determine the discriminatory power of the 27 Y-STR markers, the Investigator Argus Y-28 Kit (Qiagen) should be tested on paternally related individuals, paying special attention to the rapidly mutating Y-STR markers that this kit contains (DYS449, DYS481, DYS570, DYS576, DYS518, and DYS627).

The diversity of haplogroups identified following data analysis reflects the background and Romania’s geographical position on the route of the great migrations at the crossroads between Western Asia and Central and Western Europe, as well as between Southern and Northern Europe. The slight gradient of R1a enrichment in Moldavia and Transylvania relative to Muntenia and the higher J2a frequency in Muntenia reported here is broadly consistent with previously proposed population-history narratives for South-Eastern Europe [17], though we emphasize that STR-based haplogroup prediction alone, without single nucleotide polymorphism (SNP) subclade resolution or direct haplotype comparison against neighboring populations, cannot itself test or confirm specific migration hypotheses.

So, because haplogroup prediction relied on STR-based data rather than SNP typing the results obtained only provide a general picture of the events that led to the current structure of the Romanian population. Future work integrating Y-chromosome Single Nucleotide Polymorphism (Y-SNP) genotyping should allow more precise sub-haplogroup resolution and refined intra-population structure analysis.

## 4. Materials and Methods

### 4.1. Sample Collection

In this study we used the same sample of 928 unrelated men, born in Romania, used in the population-based study to determine allele frequencies for autosomal STR markers published the previous year [24]. We proceeded in this way to allow future comparison of the particularities observed in the analysis of autosomal STR markers with those observed in the analysis of Y-chromosome STR markers.

The 928 samples were selected with the aim of reflecting the percentages of Romania’s population for each county and Bucharest, as reported in the 2021 census. For 33 counties and Bucharest, this target was met; for 3 counties, the number of samples collected came very close to the target (over 80% of the required samples were collected); and for 5 counties, the required number of samples was not reached, with the collection rate ranging from 14% to 57%. However, the results obtained are not affected because all the analyses in this study were conducted at the national or historical-region level, where sample sizes (n = 215–405) are sufficient to support reliable diversity and frequency estimates; county-level counts are reported in Table 3 to document geographic coverage rather than to support county-level inference.

To verify the absence of close relatives among the 928 donors, a pairwise kinship analysis was performed using previously generated 21-locus autosomal STR profiles for the same sample set and population-specific allele frequencies [24]. Likelihood-ratio kinship tests (Paternity Index, Sibling Index) were computed for all 430,128 possible sample pairs. No parent–offspring pairs were detected among the 928 individuals. Two pairs showed statistically significant evidence of elevated relatedness after Bonferroni correction for multiple comparisons, and a third pair was borderline; however, cross-checking the Y-STR haplotypes of these candidates confirmed that none share a paternal lineage (haplotypes differed at 13–20 of 27 marker fields, well beyond the range expected for paternal relatives). As Y-STR haplotypes are inherited exclusively through the paternal line, this confirms that no donors in the present sample are related through the paternal lineage, and that the reported haplotype frequency and random match probability estimates are not affected by relatedness among donors.

This set of samples is represented by epithelial buccal cells collected from convicted persons, in accordance with national law number 76 of 2008, using EasiCollect^®^ Plus (Qiagen, Venlo, The Netherlands) FTA card devices (n = 897) or buccal swabs (n = 31).

### 4.2. DNA Extraction

For most samples, polymerase chain reaction (PCR) amplification was performed directly from FTA card material, without a separate conventional DNA extraction step, but for some of the samples genomic DNA extraction was performed before Y-STR PCR amplification.

Genomic DNA was extracted with the QIAamp Investigator BioRobot Kit (Qiagen) on the BioRobot Universal System (Qiagen) when samples were collected using buccal swabs; and when direct PCR amplification from the FTA card material did not yield optimal results because the collection procedure was not performed properly and the amount of biological material recovered on FTA card was low, DNA extraction was performed using the QIAsymphony DNA Investigator Kit (Qiagen) on the QIAsymphony SP/AS instrument (Qiagen).

### 4.3. DNA Quantification

The quantification of DNA was performed only for the samples extracted with the QIAsymphony DNA Investigator Kit (Qiagen) on the QIAsymphony SP/AS instrument (Qiagen) using the Investigator Quantiplex Pro RGQ (Qiagen) on the RotorGene Q Real-Time System (Qiagen) in accordance with the kit manufacturer’s protocol.

### 4.4. Y-STR PCR Amplification

The PCR amplification was performed in accordance with the kit manufacturer’s protocol using the Investigator Argus Y-28 Kit (Qiagen) and a Veriti™ Thermal Cycler (ThermoFisher Scientific, Waltham, MA, USA) which targeted 27 Y-STR loci (DYS19, DYS389I, DYS389II, DYS390, DYS391, DYS392, DYS393, DYS437, DYS438, DYS439, DYS448, DYS456, DYS458, DYS635, YGATAH4, DYS385a, DYS385b, DYS449, DYS460, DYS481, DYS518, DYS533, DYS549, DYS570, DYS576, DYS627 and DYS643), and two quality control markers.

If the presence of microvariants was observed following genotyping, they were confirmed using the Yfiler™ Plus PCR Amplification Kit (ThermoFisher Scientific) and a Veriti™ Thermal Cycler (ThermoFisher Scientific) using the kit manufacturer’s protocol.

### 4.5. Detection and Genotyping

Following amplification, electrophoresis of PCR products was carried out using the ABI 3500 Genetic Analyzer (ThermoFisher Scientific) together with allelic ladders, positive and negative controls using the recommendations of the amplification kit manufacturer.

The data collected by the 3500 Genetic Analyzer (ThermoFisher Scientific) were analyzed and the genotypes were assigned using GeneMapper ID-X 1.6 as the amplification kit manufacturer’s protocol specified.

### 4.6. Statistical Analysis

The calculations for gene diversity (GD), polymorphism information content (PIC), match probability (PM) using the counting method and power of discrimination (PD) were performed using the online program STR Analysis for Forensics (STRAF) v2.2.2 [25].

The Discrete Laplace method was used for determining the mixture model clustering, haplotype frequencies and match probability [11,12]. The computations were performed with the R package disclapmix v1.7.5 for a 17 Y-STR marker subset (DYS19, DYS389I, DYS389II, DYS390, DYS391, DYS392, DYS393, DYS437, DYS438, DYS439, DYS448, DYS456, DYS458, DYS635, YGATAH4, DYS385a and DYS385b) and also with the R package disclapmix2 v0.6.1 using all 27 Y-STR markers analyzed [13]. All computations, including disclapmix v1.7.5 and disclapmix2 v0.6.1 analysis, were performed in R v4.5.3.

The duplicated locus DYS385 was represented differently between the two model implementations: for disclapmix v1.7.5 it was entered as two ordered pseudo-loci, DYS385a (lower repeat number) and DYS385b (higher repeat number), the standard workaround required because the original DL model [11,12] assumes a single integer-valued allele per locus, whereas for disclapmix2 v0.6.1, which was purpose-built to natively support multi-copy loci, partial repeats and null alleles [13], DYS385 was instead entered as a single multi-copy field. DYS389I and DYS389II were entered as separate loci in both analyses without adjusting for their known structural dependency (the DYS389II amplicon includes the DYS389I repeat region), following the convention used in the DL method’s own reference datasets [11,12], in which loci are modeled as conditionally independent given cluster membership so that correlated multi-locus patterns are captured through cluster assignment rather than through an explicit locus-pair covariance term. Regarding differential mutation rates, the DL model does not assume equal variability across loci: it estimates an independent dispersion parameter for every locus within every cluster, fitted directly from the observed within-cluster allele-count variation [11,12].

Y-chromosome haplogroup prediction was performed with the web-based tool Athey’s Haplogroup Predictor [26] using only 25 of the 27 Y-STR markers (DYS518 and DYS627 are not included in the list of 111 markers of this tool). The criterion for designating a haplogroup to a haplotype was the highest value of the Bayesian probability returned by the prediction tool. Haplogroup diversity was estimated using Nei’s formula applied to haplogroup frequencies [27].

To evaluate whether the observed differences in haplotype composition among the three major historical regions (Moldavia, Transylvania, and Muntenia) were statistically significant, an Analysis of Molecular Variance (AMOVA) was performed using the R_ST_ estimator, which is appropriate for microsatellite loci evolving under a stepwise mutation model [22]. All AMOVA/RST calculations were implemented in Python (NumPy v2.5.1, SciPy v1.18.0).

## 5. Conclusions

This study delivers a fully characterized 27-locus Y-STR database for 928 Romanian males, representative of all counties and the three major historical regions. The use of disclapmix2 v0.6.1 across the extended marker panel not only achieved complete haplotype uniqueness but yielded match probabilities approximately six orders of magnitude lower than the 17-locus model, providing a significantly stronger evidential framework for casework. The documented haplogroup composition—led by R1a, I2a, E1b1b, J2a, and R1b—is consistent with Romania’s position as a demographic palimpsest shaped by Steppe migrations, Balkan Neolithic lineages, and Near Eastern gene flow.

The present dataset constitutes the largest geographically complete Y-STR reference resource for Romania published to date. Future work should focus on SNP-based sub-haplogroup resolution and on testing the Investigator Argus Y-28 Kit on paternally related individuals to further characterize discriminatory power within patrilineal groups.

## Figures and Tables

**Table 1:** Number of alleles per locus, gene diversity (GD), polymorphism information content (PIC), match probability (PM) using the counting method and power of discrimination (PD) for 27 Y-STR markers.

PD	PM (Counting Method)	PIC	GD	Highest Allele Found	Lowest Allele Found	No. of Unique Alleles	Locus
0.770	0.230	0.732	0.771	18	12	7	DYS19
0.831	0.169	0.810	0.832	18	9	11	DYS385a
0.802	0.198	0.776	0.803	21	10	13	DYS385b
0.509	0.491	0.458	0.509	16	10	6	DYS389I
0.754	0.246	0.714	0.755	34	26	9	DYS389II
0.724	0.276	0.680	0.725	27	19	9	DYS390
0.517	0.483	0.426	0.518	13	8	6	DYS391
0.350	0.650	0.327	0.351	16	10	7	DYS392
0.518	0.482	0.461	0.519	15	10	6	DYS393
0.586	0.414	0.507	0.586	16	13	5	DYS437
0.696	0.304	0.649	0.697	13	8	6	DYS438
0.714	0.286	0.663	0.714	15	9	7	DYS439
0.593	0.407	0.534	0.594	23	15	10	DYS448
0.834	0.166	0.814	0.835	39	25	13	DYS449
0.708	0.292	0.667	0.709	19	12	8	DYS456
0.773	0.227	0.737	0.774	22.2	13	15	DYS458
0.657	0.343	0.590	0.657	14	8	6	DYS460
0.845	0.155	0.829	0.846	32	18	15	DYS481
0.834	0.166	0.813	0.835	45	34	12	DYS518
0.620	0.380	0.570	0.621	15	8	8	DYS533
0.632	0.368	0.580	0.632	15	7	7	DYS549
0.806	0.194	0.779	0.807	25	12	12	DYS570
0.765	0.235	0.729	0.765	25	13	11	DYS576
0.864	0.136	0.850	0.865	24	14	11	DYS627
0.737	0.263	0.700	0.738	27	19	9	DYS635
0.708	0.292	0.672	0.709	14	7	8	DYS643
0.620	0.380	0.544	0.621	14	9	6	YGATAH4

**Table 2:** Y-chromosome haplogroup counts and relative frequencies (%) in the total Romanian sample and three historical regional subgroups.

Muntenia (%)	Muntenia (n)	Transylvania (%)	Transylvania (n)	Moldavia (%)	Moldavia (n)	Romania (%)	Romania (n)	Y Haplogroup
0	0	0.32	1	0	0	0.11	1	E1b1a
17.78	72	16.23	50	9.30	20	15.30	142	E1b1b
2.72	11	2.27	7	4.19	9	2.91	27	G2a
5.68	23	7.14	22	4.65	10	5.93	55	I1
15.80	64	15.26	47	18.14	39	16.16	150	I2a
2.72	11	1.95	6	2.79	6	2.48	23	I2b
1.73	7	1.30	4	1.86	4	1.62	15	J1
17.78	72	12.34	38	14.42	31	15.19	141	J2a
3.70	15	3.57	11	3.26	7	3.56	33	J2b
0	0	0.32	1	0.47	1	0.22	2	L
0.99	4	1.30	4	2.79	6	1.51	14	N
0.25	1	0	0	0	0	0.11	1	Q
16.05	65	23.70	73	23.26	50	20.26	188	R1a
13.58	55	13.96	43	13.49	29	13.69	127	R1b
0.25	1	0	0	0.47	1	0.22	2	R2
0.99	4	0.32	1	0.93	2	0.75	7	T
100.00 *	405	100.00 *	308	100.00 *	215	100.00 *	928	Total
0.8610 ± 0.0248		0.8518 ± 0.0291		0.8584 ± 0.0338		0.8608 ± 0.0164		Haplogroup diversity

* Percentages are rounded to two decimal places and do not sum to exactly 100% due to rounding.

**Table 3:** Distribution of analyzed samples.

County	Historical Region	Number of Analyzed Biological Samples
Bacău	Moldavia	32
Botoșani	Moldavia	21
Galați	Moldavia	26
Iași	Moldavia	40
Neamț	Moldavia	24
Suceava	Moldavia	34
Vaslui	Moldavia	20
Vrancea	Moldavia	18
Alba **	Transylvania	7
Arad	Transylvania	22
Bihor	Transylvania	29
Bistrița-Năsăud	Transylvania	16
Brașov	Transylvania	29
Caraș-Severin	Transylvania	13
Cluj	Transylvania	36
Covasna	Transylvania	10
Harghita *	Transylvania	13
Hunedoara	Transylvania	19
Maramureș	Transylvania	24
Mureș	Transylvania	27
Sălaj	Transylvania	11
Satu-Mare **	Transylvania	4
Sibiu	Transylvania	20
Timiș *	Transylvania	28
Argeș	Muntenia	30
Brăila	Muntenia	15
Bucharest	Muntenia	90
Buzău	Muntenia	21
Călărași **	Muntenia	5
Constanța	Muntenia	34
Dâmbovița	Muntenia	25
Dolj *	Muntenia	30
Giurgiu **	Muntenia	8
Gorj	Muntenia	17
Ialomița	Muntenia	13
Ilfov **	Muntenia	4
Mehedinți	Muntenia	12
Olt	Muntenia	20
Prahova	Muntenia	36
Teleorman	Muntenia	17
Tulcea	Muntenia	10
Vâlcea	Muntenia	18

* Counties with number of samples very close to the target (>80%); ** Counties with number of samples not reached (14–57%).

## Data Availability

The original contributions presented in this study are included in the article and Supplementary Materials. Further inquiries can be directed to the corresponding authors.

## Supplementary Materials

The following supporting information can be downloaded at: https://www.mdpi.com/article/10.3390/ijms27167121/s1.

## Abbreviations

The following abbreviations are used in this manuscript:

BIC	Bayesian Information Criterion
DL	Discrete Laplace
DNA	Deoxyribonucleic Acid
EU	European Union
GD	Gene diversity
HGD	Haplogroup diversity
PCR	Polymerase Chain Reaction
PD	Power of discrimination
PIC	Polymorphism information content
PM	Match probability
SNP	Single Nucleotide Polymorphism
STR	Short Tandem Repeat
STRAF	STR Analysis for Forensics
Y-SNP	Y-chromosome Single Nucleotide Polymorphism
Y-STR	Y-chromosome short Tandem repeat
