# Supplementary material for: The Genetic Structure of the Romanian Male Population Revealed by 27 Y-Chromosome STR Loci
Source: Int J Mol Sci. 2026 Aug 8;27(16):7121. doi: 10.3390/ijms27167121 (PMC13513713; doi:10.3390/ijms27167121)
Supplement: Supplementary file 1 [file ijms-27-07121-s001.zip › Figure S1_romania_haplogroup_map.html]

Loading Romania map data…

Pie size ∝ sample size · hover slices for details · county borders from real geographic data
